# Strategy for Managing Industrial Anaerobic Sludge through the Heterotrophic Cultivation of *Chlorella sorokiniana*: Effect of Iron Addition on Biomass and Lipid Production

**DOI:** 10.3390/bioengineering8060082

**Published:** 2021-06-10

**Authors:** Esteban Charria-Girón, Vanessa Amazo, Daniela De Angulo, Eliana Hidalgo, María Francisca Villegas-Torres, Frank Baganz, Nelson. H. Caicedo Ortega

**Affiliations:** 1 Departamento de Ingeniería Bioquímica, Facultad de Ingeniería, Universidad Icesi, Calle 18 No. 122–135, Cali 760031, Colombia; charria21@gmail.com (E.C.-G.); vaneaw_806@hotmail.com (V.A.); daniela.deangulo@gmail.com (D.D.A.); ely.hidalgo999@gmail.com (E.H.); 2 Departamento de Ciencias Químicas, Facultad de Ciencias Naturales, Universidad Icesi, Calle 18 No. 122–135, Cali 760031, Colombia; mfvillegas@icesi.edu.co; 3 Centro BioInc, Universidad Icesi, Calle 18 No. 122–135, Cali 760031, Colombia; 4 Department of Biochemical Engineering, University College London, Gordon Street, London WC1H 0AH, UK; f.baganz@ucl.ac.uk

**Keywords:** anaerobic sludge, chlorella sorokiniana, heterotrophic cultivation, Iron, modeling

## Abstract

Microalgae provides an alternative for the valorization of industrial by-products, in which the nutritional content varies substantially and directly affects microalgae system performance. Herein, the heterotrophic cultivation of *Chlorella sorokiniana* was systematically studied, allowing us to detect a nutritional deficiency other than the carbon source through assessing the oxygen transfer rate for glucose or acetate fermentation. Consequently, a mathematical model of the iron co-limiting effect on heterotrophic microalgae was developed by exploring its ability to regulate the specific growth rate and yield. For instance, higher values of the specific growth rate (0.17 h^−1^) compared with those reported for the heterotrophic culture of *Chlorella* were obtained due to iron supplementation. Therefore, anaerobic sludge from an industrial wastewater treatment plant (a baker’s yeast company) was pretreated to obtain an extract as a media supplement for *C. sorokiniana*. According to the proposed model, the sludge extract allowed us to supplement iron values close to the growth activation concentration (K_Fe_ ~12 mg L^−1^). Therefore, a fed-batch strategy was evaluated on nitrogen-deprived cultures supplemented with the sludge extract to promote biomass formation and fatty acid synthesis. Our findings reveal that nitrogen and iron in sludge extract can supplement heterotrophic cultures of *Chlorella* and provide an alternative for the valorization of industrial anaerobic sludge.

## 1. Introduction

During the past decades, industrialization has led to large volumes of generated industrial wastewater and high-organic content waste worldwide [1]. Despite advances in wastewater treatment strategies, the final disposal of the sludge generated during treatment remains a critical problem. Although anaerobic sludge is produced in lower quantities than aerobically produced sludge, both are commonly managed and disposed of in sanitary landfills or incinerated [2,3].

However, with these procedures, the contamination and supersaturation of land and water bodies have caused adverse environmental effects [2,3]. Therefore, the use of these by-products in other applications for economic gain and environmental protection is desirable. A promising alternative includes sludge valorization using microalgae biorefinement processes, which could help to recover or reuse waste and industrial by-products and achieve sustainable biomass production of microalgae for diverse purposes.

Several photosynthetic microalgae have gained attention as promising candidates for the sustainable production of high added-value products, including lipids, lutein, β-carotene, astaxanthin, polysaccharides, and even antibiotics [4]. *Chlorella* is among the most promising genera from an industrial perspective due to its simple, single-celled structure, ease of harvest; efficient photosynthetic yield; high growth rate; and lipid production of over 50% dry-cell weight. *Chlorella* is commercially marketed as a food supplement and reports worldwide sales higher than 38 billion dollars [5]. 

From the biodiesel production perspective, lipids from its heterotrophic cultivation are considered a more viable feedstock for biodiesel production due to the high production yields of total and neutral lipids [6]. *Chlorella sorokiniana* has been used for industrial water waste management because of its ability to grow in the presence of high organic content [7,8]. It is also increasingly commercialized, mainly in the US, as a dietary supplement for weight control and protection against cancer [9].

Due to the high cost of microalgae cultivation, only a few microalgal species have managed to position themselves in the industry [10]. Compared to photoautotrophic and mixotrophic growth, one of the significant advantages of heterotrophic growth is achieving higher biomass productivity over standard photoautotrophic systems. Additional benefits of heterotrophic cultivation include a significant decrease in photobioreactor operational time and occupancy area used in scaling-up processes [11]. Although the above demonstrates a path to large-scale production using microalgae, the high cost of biomass production must be addressed to become economically feasible [12]. 

A potential strategy to reduce biomass production costs is to replace some nutrient sources in culture media with anaerobically digested waste sludge (ADWS) [13]. ADWS, supplemented with volatile acids, was shown to produce a higher quantity of microalgal biomass than nitrogen and phosphate from traditional sources under mixotrophic growth conditions [14]. However, ADWS from industrial processes brings additional challenges due to the limited composition and potential contaminants. Even so, a recent study demonstrated that after pretreatment, the use of synthetic wastewater supplemented with tetrachlorophenol for *Chlorella vulgaris* growth under mixotrophic conditions promoted starch production [13].

Industrial wastewater plants (IWWPs) use ferric chloride to control noxious odors from volatile compounds, such as sulfur [15]. This practice is conducive to the enrichment of anaerobic sludge with ferric ions. Iron is of interest as it is involved in many functions, including metabolic regulation, cellular synthesis, chlorophyll synthesis (photosynthesis), respiration, intracellular transport (ion transfer), and protein and lipid biosynthesis [16]. In recent years, the effect of iron on microalgae growth, mainly photoautotrophic growth, has been the subject of several studies providing insights into the effects of iron on growth and lipid biosynthesis in a light-dependent metabolism [17]. 

The supplementation of this metal may lead to higher growth and lipid production rates, mainly by improving the photosynthetic capacity [18]. This study provides an opportunity to thoroughly evaluate the relationship between heterotrophic cultivation and iron as an alternative to improve the performance of microalgal biomass production. Moreover, apart from the mathematical model of the effect of iron on lipid accumulation for autotrophic microalgae proposed by Concas et al. [19], no knowledge of mechanistic modeling of iron’s effect on microalgal metabolism has been reported; thus, iron’s kinetic effect has been overlooked.

We evaluated the impact of iron on the heterotrophic cultivation of *C. sorokiniana* and the use of anaerobic sludge extract from the industry as an iron source. Nutrient co-limitation was observed by monitoring oxygen transfer rate (OTR) in shake-flask cultures. Then, the effect of iron supplementation on microalgal growth was assessed. Microalgae growth was modeled and simulated under batch and fed-batch conditions accounting for iron’s effect on the growth rate and substrate yield. Finally, the effects of different sludge extract dilutions were evaluated on microalgae growth and lipid production during nitrogen-starved heterotrophic culture as a proof of concept for industrial ADWS valorization.

## 2. Materials and Methods

### 2.1. The Heterotrophic Cultivation of C. sorokiniana

The *C. sorokiniana* strain UTEX 1602 used in this study was obtained from the University of Texas collection (Austin, TX, USA). This strain was maintained at 4 °C in Tris-acetate–phosphate (TAP) solid media supplemented with 5 g L^−1^ glucose [20]. Heterotrophic cultures were set up with 300 mL of TAP medium into 500 mL stirred-tank mini-bioreactors (Applikon^®^-Biotechnology, JG Delft, The Netherlands) and were conducted by duplicated. The cultivation conditions were as follows: 250 rpm, 30 °C, oxygen in excess (2 v.v.m), and constant pH (7.5) adjusted with HCl (1 M) and NaOH (1 M). Culture axenicity was checked through all the set-up fermentations, including the inoculum development, by counting the algal cell number by microscope (Counting chamber, Neubauer improved, BLAUBRAND^®^, CE-IVD, Wertheim, Germany), and checking contaminants by Gram staining combined with the plating of broth samples in selective media for moulds and bacteria.

### 2.2. Quantification of Respiratory Activity in Shake Flasks

The physiological state and oxygen-dependent metabolic activity of *C. sorokiniana* were assessed in shaken flasks (250 mL) using an online-OTR measurement device (RAMOS, Kuhner^®^, Birsfelden, Switzerland). Two organic carbon sources, glucose and acetate, were evaluated using a factorial experimental design 2^2^ in concentrations within the range of optimal values for *Chlorella* grown under heterotrophic conditions [4], as shown in Table 1. Each experimental unit was filled with 10 mL TAP medium (1.0 mL Hutner’s trace metals, 25 mL salts stock, and 0.375 mL phosphate solution) [20] in with phosphate buffer (1 M), and 10% *v v*^−1^ of three days cultivated inoculum (starved in exponential growth phase). The cultivation conditions were as follows: 250 rpm, 30 °C, oxygen in excess (2 v.v.m), and initial pH (7.5) adjusted with a sulphuric acid solution. These Erlenmeyer flasks for RAMOS shaker were covered at the top with a sealed cap with the respective membranes for selective O_2_ diffusion and a partial pressure O_2_ sensor.

### 2.3. Evaluation of the Iron Effect on Heterotrophic Cultivation

The effect of ferric ion (Fe^3+^) supplementation on *C. sorokiniana* growth was evaluated by adding two concentrations of FeCl_3_·6H_2_O (12 and 60 mg L^−1^) based on reported values during the late exponential phase of batch cultures [21]. This state was fixed when the metabolic activity decreased according to the OTR assessment, which also corresponded to the moment before the specific growth rate decreased below 0.13 h^−1^. Simultaneously, a trial with no addition was used as a control treatment. Iron was supplied from a stock solution (FeCl_3_·6H_2_O + EDTA) at a concentration of 4.83 g L^−1^. The TAP media had a baseline concentration of 5.4 mg L^−1^ FeCl_3_·6H_2_O.

### 2.4. Kinetic Model for Iron Co-Limitation Effect

The kinetic model for microalgae growth was developed using a Monod-type model (Equation (1)) to describe the combined limitations of glucose and iron on the heterotrophic growth of *C. sorokiniana*. The kinetic parameters, maximum specific growth rate (*µ_max_*), affinity constant for glucose (*K_s_*), and substrate yield (*Y_x/s_*), were determined from experimental batch data obtained in Section 2.3. Figure S1 (see in Supplementary Materials) presents all the details of the methodology for this co-limitation model as a flowchart.

Furthermore, iron was assumed to modulate the maximum growth rate (*µ_max_*) and substrate yield (*Y_x/s_*). From this and taking into account iron inhibition pattern on microalgal growth [22], a new maximum specific growth rate (*µ_Fe_*) was considered under an uncompetitive kinetic model Equation (1b). Moreover, a modification on substrate yield (*Y_x/s_*) as a correlation of maximum growth rate change due to iron (Fe) was represented as *Y_x/s, Fe_*, which was defined as a function of iron affinity constant (*K_Fe_*) and iron substrate modulation constant (*n*) in Equation (2). 

Thus, allowing a generalization for different microalgae species, which have significantly different affinities for this nutrient, and different inhibition patterns represented by the iron inhibition constant (*Ki_Fe_*). Equations (1) and (3) describe the growth and substrate (S) consumption kinetics used in this model, respectively. Additionally, Equation (4) describes the iron consumption kinetics as a function of the iron and biomass concentrations, where *P*_Fe_ represents a first-order constant. The kinetic parameters corresponding to the representation of the iron co-limiting effect were estimated from experimental data of cultures supplemented with iron during the late exponential phase obtained in the experiment from Section 2.3.

(1) $$\frac{dX}{dt}=\mu X=\frac{\mu_{Fe}.S}{Ks+S}X\;=\frac{\mu_{Fe}.Fe.S.X}{\left(K_{Fe}+Fe+\frac{{Fe}^{2}}{Ki_{Fe}}\right)\left(Ks+S\right)}$$

(2) YXS,Fe=(n×(μFeμmax)KFe)YXS

(3) dSdt=−μXYXS,Fe=−μFe(1−KFe).μmax.Fe.S.X(nYXS)(KFe+Fe+Fe2KiFe)(Ks+S)

(4) $$\frac{dFe}{dt}=-P_{Fe}.Fe.\mu X=-\frac{P_{Fe}.Fe.\;\mu_{Fe}{}^{\left(1-K_{Fe}\right)}\mu_{\max}.Fe.S.X}{\left(K_{Fe}+Fe+\frac{{Fe}^{2}}{Ki_{Fe}}\right)\left(Ks+S\right)}$$

Subsequently, the co-limitation model was validated against the batch experimental data of iron-supplemented cultures of *C. sorokiniana* as shown in Section 2.3, together with the reported data obtained from Ren et al. [22]. They evaluated the effect of different metals, including iron, on the heterotrophic growth of *Scenedesmus* sp.

Afterward, a bioaugmentation process (fed-batch) was simulated based on the co-limitation kinetic model with glucose as carbon source and a single iron addition at the end of exponential growth (15 h) in the previous batch culture achieving a final FeCl_3_·6H_2_O concentration of 60 mg L^−1^. Next, a glucose stock solution (14.7 g L^−1^) was estimated to correspond to an exponential feeding profile of 6 h, starting with an initial flow rate of 0.23 mL min^−1^ and a fixed growth rate of 0.13 h^−1^. All the details of the ODE mass balances for this exponential fed-batch are presented in Table S1 (see in Supplementary Materials). All simulations were performed using the numerical software MATLAB^®^ 2019b (The MathWorks, Inc.) with ODE45 solver (the Runge–Kutta method), and the model parameters were calibrated by using the *fminsearch* function to minimize the sum squared errors (SSE < 0.05) between the predicted and experimental data.

At the same time, an experimental set-up was performed with the same profile of glucose feeding and the single 60 mg L^−1^ iron addition. A carbon source limitation was guaranteed experimentally by adding NH_4_Cl during the fed-batch process, ensuring that it was 1.2-times the required amount for the expected biomass production. Several samples were taken for biomass monitoring during the whole process.

### 2.5. Use of Anaerobically Digested Waste Sludge Extract as a Media Supplement for C. sorokiniana Cultivation under Heterotrophic Conditions

A second heterotrophic culture of *C. sorokiniana* was conducted to identify the best strategy to supplement anaerobically digested sludge extract and simultaneously promote lipid production. Sludge was obtained from the wastewater treatment plant (WWTP) of the baker’s yeast company Levapan S.A, Tuluá, Valle del Cauca, Colombia. First, anaerobic sludge was pretreated, adding 0.1 g NaOH per gram of sludge and heating at 121 °C for 15 min. Then the pH was adjusted to 7.0 with 98% H_2_SO_4_, and homogenized using mechanical stirring for 24 h. 

Finally, this pretreated sludge solution was centrifuged (Hettich^®^, Kirchlengern, Germany) at 3800 rpm for 15 min, and the final supernatant was filtered consecutively through 8- and 0.21-μm membranes. The final filtrate corresponded to the sludge extract (10% *v v*^−1^), which contained the sludge-derived nutrients. This extract was used as a stock solution for supplementing heterotrophic cultures of *C. sorokiniana*.

The effect of sludge extract supplementation was assessed after a series of adjustments in TAP media, which were made to assure the optimum conditions for lipid production. These included selenium and cobalt, Na_2_SeO_3_ (0.035 mg L^−1^) and CoCl_2_∙6H_2_O (0.49 g L^−1^), respectively [23,24]. Additionally, the medium was nitrogen-deprived (not NH_4_Cl added) to promote lipid accumulation [10]. For each experimental set-up, successive glucose additions were made to maintain a concentration between 5 and 10 g L^−1^. 

Similarly, sludge extract additions, which provided iron, carbon, and nitrogen sources, among other nutrients, were evaluated to setback the oxygen dependant activity, which was deduced from monitoring the dissolved oxygen concentration in the medium. Hence, high values of pO_2_% (>70%) indicated no or low-active cellular metabolic activity [25].

### 2.6. Analytical Methods

After broth sample centrifugation and washing, the microalgal biomass was measured as the optical density at 741 nm (OD_741_) and converted to dry weight (DW) using a linear regression correlation. The medium supernatant obtained after sample centrifugation (7000 rpm, 10 min, Hettich^®^, Kirchlengern, Germany) was used to quantify the carbon and nitrogen sources. Glucose was quantified using the K-GLUC D-Glucose Assay Kit (GOPOD Format, Megazyme^®^, Bray, Ireland), and ammonium was quantified using the K-AMIAR Ammonia Assay Kit (Rapid) (Megazyme^®^, Bray, Ireland). 

All spectrophotometric readings were performed using a multimode microplate reader (Varioskan™LUX, Thermo Fisher Scientific, Waltham, MA, USA.). The lipid content was estimated during the culture using a colorimetric method with the lipophilic reagent Nile red, allowing the direct quantification of lipids and using a correlation obtained with canola oil as a standard [26]. All fluorometric measurements were performed on a Varioskan™LUX fluorescence spectrometer (Thermo Fisher Scientific, Waltham, MA, USA) with temperature control set at 40 °C and 300 rpm.

### 2.7. Statistical Analysis

All the results are reported as an average of the two experimental values obtained. OriginPro 2020b was used to prepare the graphs and calculate the average values and standard deviations between biological replicates.

## 3. Results and Discussion

### 3.1. Quantification of Respiratory Activity in Shake Flasks

**Figure 1:**
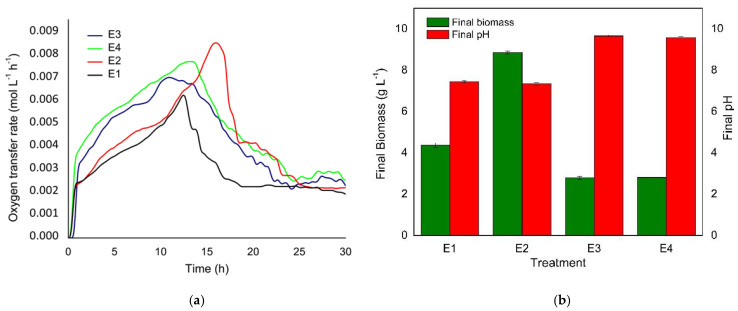
(**a**) Online measurement of the oxygen transfer rate (OTR) of *C. sorokiniana* heterotrophic cultures grown in TAP medium supplemented with different carbon sources (E1 and E2 with glucose, E3 and E4 with acetate and glucose). (**b**) Final biomass concentration and medium-pH reached for each treatment.

During approximately the first 13 h of the heterotrophic *C. sorokiniana* culture, treatments with glucose and acetate (E3 and E4) displayed higher biological activity than those with glucose alone (Figure 1a). Subsequently, for glucose treatment (E1), a significant reduction in biological activity was observed through its respirometric assessment between 12 and 15 h showing a substantial decrease in the OTR until 18 h of fermentation when it reached a plateau. This latter was reached for glucose treatment (E2, 10 g L^−1^) after 25 h, indicating a nutrient limitation other than the carbon source, according to the interpretation of data exposed by this device [27,28].

Increases of 28.4% and 28.1% in the pH values were achieved for the E3 and E4 treatments compared to their initial condition, respectively, which can be explained due to the acetate metabolism in microalgal cells [4]. Similarly, this physiological response led to a final biomass concentration (Figure 1b) for both treatments (2.8 g L^−1^) lower than E1 and E2 at 4.4 and 8.8 g L^−1^, respectively. Based on the above, both carbon sources significantly affected the final biomass value (*p* < 0.05); however, only the acetate affected the pH significantly compared to the initial condition. 

According to the OTR course, the E3 and E4 treatments showed the highest biological activity at the beginning of cultivation, directly related to carbon source preference for acetate compared with glucose when both are present [29,30]. However, in this study, all treatments with 5 g L^−1^ acetate prompted an uncontrolled pH change under a buffered-media condition. This physiological response, caused by the rapid conversion of acetate to acetyl-CoA, requires pH-auxostat fed-batch strategies to avoid growth inhibition [31].

These results showed that the co-fermentation of acetate and glucose by the *C. sorokiniana* cells induced a net consumption of H^+,^ elevating the pH from optimal biomass formation. Only under the fermentation condition of glucose, did the TAP medium composition seem limited at the end of the batch cultivation by nutrients other than the carbon source.

### 3.2. Evaluation of the Iron Effect on Heterotrophic Cultivation

Iron, an essential nutrient for all organisms, is associated with photoautotrophic microorganisms’ electron transport chains and photosynthetic performance, including microalgae [32]. Additionally, iron deficiencies have been extensively studied in photoautotrophic and mixotrophic microalgae cultivation [19]. Furthermore, *Chlorella* studies have focused on the impact of iron supply on biomass and lipid production for photosynthetic metabolism and understanding iron transport and uptake at the subcellular level. A study reported that, for photoautotrophic cultures of *C. sorokiniana*, the addition of 2.7 mg L^−1^ iron at the beginning of cultivation extended the exponential growth phase and increased the lipid accumulation three-fold compared with the control [17]. 

On the other hand, Ren et al. [22] studied the effects of different metal ions, for instance iron, biomass, and lipid accumulation, in *Scenedesmus* sp. under heterotrophic culture conditions. They demonstrated that iron deficiency had an essential role in achieving the optimal biomass productivity in heterotrophic cultures, similar to light-dependent microalgae cultures [19]. In our opinion, the iron effect on heterotrophic microalgae growth has not been studied in depth for major species. For example, for the model microalga *Chlamydomonas reinhardtii*, the relationship between heterotrophic metabolism and iron nutrition has not been extensively researched [18].

**Figure 2:**
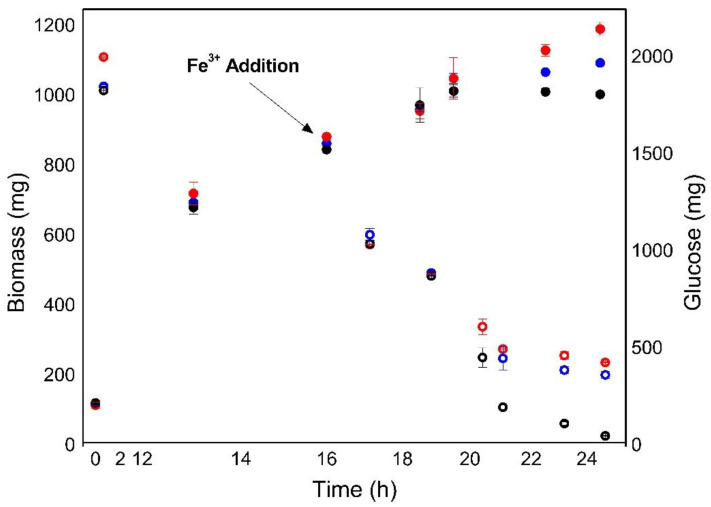
Effect of iron on *C. sorokiniana* growth in heterotrophic batch culture (medium TAP) with glucose as carbon source. Solid symbols (biomass), open symbols (glucose). Black: zero addition of Fe^3+^, Blue: 12 mg L^−1^ Fe^3+^, and Red: 60 mg L^−1^. Error bars represent standard error (*n* = 2).

Given the evidence of a nutrient limitation through respirometric assessment, we evaluated the effect of iron supplementation on the late exponential phase of heterotrophic cultures of *C. sorokiniana*. As depicted in Figure 2, iron was added to the cultures without carbon starvation (~30% *K_s_*). After 7 h of addition, treatments supplemented with 60 and 12 mg L^−1^ FeCl_3_·6H_2_O continued growing exponentially, while the control reached the stationary phase at the 19th h. Therefore, the specific growth rate increase was recalculated for each treatment between 18 and 25 h in comparison with the control (Figure S2, see in Supplementary Materials), and a significant effect on growth rate (*p* < 0.05) was observed due to iron addition. 

Then, a quadratic adjustment between the specific growth rate and added iron concentration was obtained in the range of 0–60 mg L^−1^. Higher values of added iron up to 120 mg L^−1^ were inhibitory for *C. sorokiniana* (data not shown) in our experiments. Consistently, Ren et al. [22] reported that excessively high concentrations (120 mg L^−1^) of chelated iron had an inhibitory effect on growth and lipid biosynthesis in a heterotrophic culture of *Scenedesmus* sp. Notably, iron stimuli on growth and lipid production differ between various microalgal strains, suggesting that optimum levels of Fe^3+^ are strain-dependent and need to be investigated individually for each strain.

Additionally, the iron effect on *C. sorokiniana* under heterotrophic conditions also resulted in a more efficient use of the carbon source, as depicted in Figure 2, which allowed iron supplemented cultures to obtain a higher final biomass concentration compared with other sources for non-supplemented cultures. These results suggest that iron deficiency in the culture media formulation was alleviated after iron supplementation. Combined with the initial limiting nutrient consumption (carbon source), this can produce a co-limitation effect on *C. sorokiniana* heterotrophic growth.

### 3.3. Kinetic Model for Iron Co-Limitation Effect

**Figure 3:**
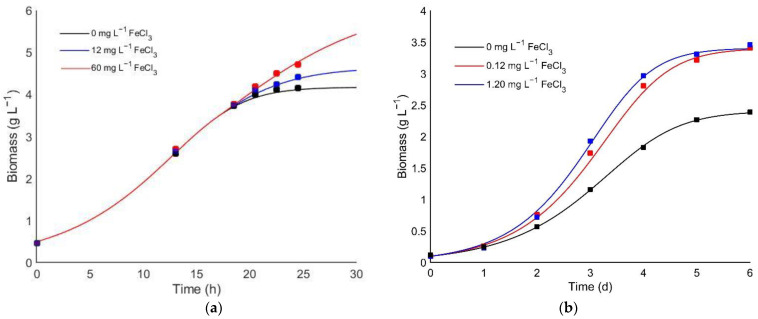
Kinetic model validation for (**a**) the iron supplementation effect during the late exponential phase of *C. sorokiniana* heterotrophic batch culture for biomass production; (**b**) the adjustment and validation for iron-supplemented cultures of *Scenedesmus* sp. reported data obtained from Ren et al. [22].

Based on iron effect supplementation data, a mathematical kinetic model representing the role of iron as a co-limiting growth nutrient was developed for the first time for heterotrophic microalgae culture. In the absence or deficiency of iron, the Monod kinetic model successfully represents growth dynamics with glucose as a limiting nutrient. However, once iron was added to the system, there was evidence of growth enhancement or inhibition depending on the supplemented concentration. Figure 3a shows an accurate model prediction of our experimental data, where it accurately fits the growth behavior once iron is added during exponential growth. 

The model fitting for experimental data was also done from an iron study on *Scenedesmus* sp. to evaluate the proposed model’s reliability to represent the co-limiting effect of this nutrient for different heterotrophic microalgae species. Thus, demonstrating that the model could accurately predict the growth kinetics for iron concentrations for cases where there is none or low inhibition (Figure 3b). This co-limiting model provided an accurate fit for experimental data describing both species’ growth kinetics (Figure 3), and the residual error between the simulated and experimental data was not significant (*p* > 0.05). The optimized kinetic parameters for the proposed model are summarized in Table 2.

Furthermore, *Scenedesmus* sp. presents a stronger affinity for iron compared with *C. sorokiniana* since the first had a greater *μ_Fe_*:*K_Fe_* ratio than the one obtained for *C. sorokiniana* [33]. On the other hand, this latter species reached a maximum growth rate of 0.256 h^−1^, which might be doubled in the absence of iron deficiency (*µ_Fe_* = 0.58 h^−1^). Therefore, due to the exhibited inhibition constant (*Ki_Fe_* = 74 mg L^−1^), *C. sorokiniana* would grow at mild iron concentrations.

In contrast, for *Scenedesmus,* a higher sensitivity (*Ki_Fe_* = 52 mg L^−1^) to iron would allow a best growth performance at low iron concentrations (Figure 3b). Krichen et al. [33] performed similar modeling approaches to investigate the direct effect of facilitation between two algal strains during the colonization phase in high-rate algal ponds (HRAP). They demonstrated that microalgae’s first colonization under a severe chemical condition arose from the rapid growth of pioneer species, such as *C. sorokiniana*. This situation facilitated the subsequent colonization of low growth specialists, such as *Scenedesmus pectinatus*. The fast depletion of the total available ammonia nitrogen could favor the specialist species growth, which is initially inhibited by free ammonia toxicity. This last suggests a direct relationship between the kinetic and environmental conditions for optimal microalgal growth.

Considering the systematic study of the heterotrophic growth of *C. sorokiniana*, a bioaugmentation stage was initiated with a batch incorporating a glucose concentration (10 g L^−1^), doubling the value estimated experimentally for the substrate affinity constant (Ks) (5.33 g L^−1^). The single addition of iron was executed at 15 h of cultivation at a concentration of 60 mg L^−1^, which is still in the growth activation range (<*Ki_Fe_*) according to the kinetic parameters. After 16 h of batch culture, coinciding with the observed respirometric data, where the OTR value started to decrease at this point (Figure 1a), and the fed-batch was started with an exponential glucose feeding. 

**Figure 4:**
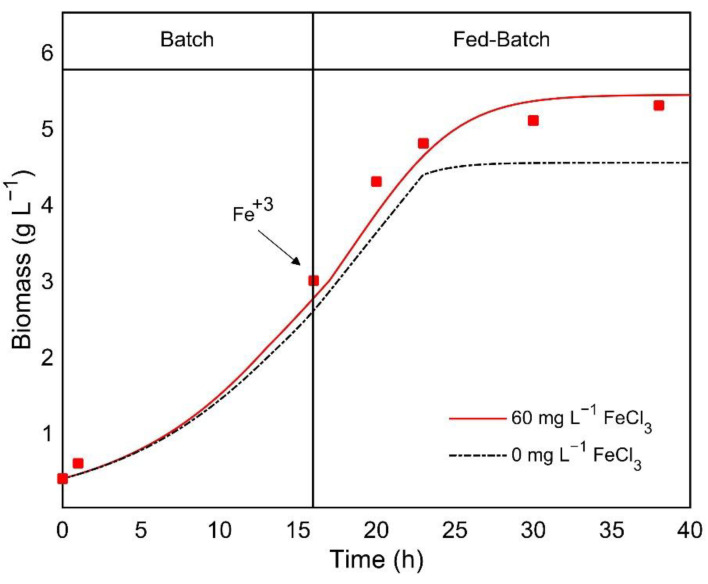
Effect of iron supplementation after 15 h on biomass formation using a feeding profile (glucose) with an exponential stepwise increased flow under heterotrophic fed-batch cultivation of *C. sorokiniana*. The red line and squares represent simulated and experimental data, respectively. The Black dotted line represents simulated data for a non-iron supplemented culture with the same feeding profile.

Figure 4 shows a comparison of simulated and experimental data, providing an accurate prediction according to the ANOVA analysis of the residual error (*p* > 0.05) (Figure 4). This indicates a higher biomass formation than with the iron-deficient model, which allowed us to achieve a specific growth rate of 0.17 h^−1^ in the exponential growth phase. Zheng et al. [5] used similar fermentation strategies for the same strain under heterotrophic conditions, with a maximum specific growth rate of 0.13 h^−1^. Thus, the specific growth rate obtained during this study resulted in a considerable improvement (31% increase) of heterotrophic achieved growth rate. Additionally, this improvement is evident compared with the maximum growth rates reported (0.1 h^−1^) for other microalgae species [4].

### 3.4. Use of Anaerobically Digested Waste Sludge Extract as a Media Supplement for C. sorokiniana Cultivation under Heterotrophic Conditions

Table 3 summarizes the elemental composition of sludge before pretreatment and the final composition of the sludge extract. Among all the components, iron is of interest because, in sludge stabilization during and after wastewater treatment, chemicals are used to remove undesirable odors, reduce volatile compounds, inactivate pathogens, and neutralize and flocculate the pollutant load. Hydrogen sulfide is removed from treated water by the in-situ addition of iron salts or oxides, such as FeCl_3_, which explains the Fe^3+^ content in both raw and pretreated sludge extract.

The concentration of TOC recovered (1178 mg L^−1^) in the sludge extract after pretreatment was higher than that reported for other treatment methods, such as the anaerobic digestion of domestic sludge [14]. This fact can be affected by the differences in each matrix’s initial TOC content and other components, which is clearest observed in TN’s concentration accomplished by Tan et al. [14], which is approximately half of the value achieved in this study (597.6 mg L^−1^). Additionally, Chen et al. [13] obtained a TOC of 268 mg L^−1^ in their synthetic industrial sludge extracts. These variations in final nutrient concentration must be considered before it is used for microalgae cultivation, depending on the species and culture conditions (mixotrophic or heterotrophic) to avoid growth inhibition [14].

**Figure 5:**
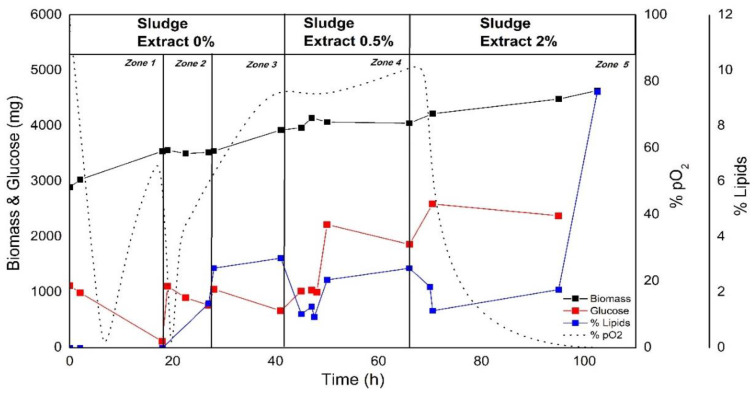
Lipid production, substrate consumption, and biomass formation profiles based on the addition of different proportions (0, 0.5, and 2% *v/v*) of anaerobic sludge extract and a bolus feeding of glucose at different cultivation times. Each zone represents a different physiological response of *C. sorokiniana* under heterotrophic culture conditions.

Pre-cultivated microalgal biomass harvested from the previously described bioaugmentation process was used as an inoculum to evaluate anaerobically digested waste sludge extract as a media supplement. Different dilutions (% *v v*^−1^) were added and assessed for their effect on biomass production, fatty acid synthesis, and dissolved oxygen consumption. At first, no sludge extract was added (Figure 5, zone 1); the medium was nitrogen-deprived, and the carbon source was added depending on the cellular requirement, avoiding cellular stress due to carbon source starvation.

During the first 18 h (zone 1), an active metabolic state was observed, supported by a substantial decrease in the dissolved oxygen partial pressure (pO_2_), which corresponds to a constant cell growth during this period (Figure 5). Even though the medium was nitrogen deprived, biomass production was not interrupted at this early stage. Between 19 and 25 h (Figure 5, zone 2), a possible alteration in the cell metabolism in response to carbon and nitrogen limitation occurred, resulting in a stagnation of biomass formation. This fact suggests a stress condition in which fatty acid synthesis was further promoted (Figure 5). Moreover, the curse of oxygen consumption suggested a decrease in respiratory cell activity. Therefore, the cells achieved a condition that enhanced lipid synthesis, which corresponds with the physiological response of *C. sorokiniana* to nitrogen starvation regardless of cultivation modes [10].

After the apparent activation of fatty acid biosynthesis, glucose was added (28 h) to maintain the carbon flow in the direction of lipid synthesis. However, lipid production was attenuated, whereas biomass production continued. According to Perez-Garcia et al. [4], the decomposition of organelles, such as chloroplasts, occurs under nitrogen insufficiency to salvage cellular nitrogen for survival. Consistently, a color change was observed during microalgal growth upon nitrogen deprivation.

Furthermore, the pO_2_ curve in this time interval shows that the oxygen supply was higher than the demand by microalgae cells. The value of pO_2_ increased until it reached a plateau close to 80%, suggesting metabolic activity consistent with low oxygen consumption (a state of starvation). Once this state was reached, the addition of sludge extract (0.5% *v* *v*^−1^) was evaluated at 46 h after readjusting the glucose concentration to 10 g L^−1^. As seen in zone 4 (Figure 5), this addition did not produce any adverse effect on biomass production; however, a partial reduction of the accumulated lipids and dissolved oxygen occurred. 

This partial reactivation of respiratory metabolism could be explained by the bioavailable nitrogen from the supplemented sludge extract. Cheah et al. [34] reported similar results during their nitrogen resupply study of *Chlorella* sp. on starved cultures. They emphasized that the lipids produced in this condition affected the synthesis of structural membrane components due to the energy obtained from their oxidation. Although nitrogen replenishment resulted in an apparent change leading to cellular recovery, the nitrogen provided by this sludge (12.5 mg TN L^−1^) addition failed to provide a lengthy response, i.e., it was not enough to overcome the starving condition. There was enough carbon source excess due to the glucose addition at culture hour 47 (C:N = 320).

The addition of fresh sludge extract was made at 70 h, at a proportion of 2% *v/v*, which corresponded to 50 mg TN L^−1^, followed by the addition of glucose to afford a concentration of 10 g L^−1^. Consequently, this promoted a more significant impact at the physiological level, as seen by the abrupt drop in pO_2_ in zone 5 (Figure 5). Simultaneous biomass and lipid production was observed after sludge extract supplementation, with an increase in lipid production rate during approximately 30 h (zone 5), leading to an increase of up to 9% (*w/w*) in total lipids, which represents a three-fold increase compared to the value reached after the previous addition of 0.5% *v v*^−1^ of the extract. 

This response is explained mainly by the nutritional contribution of the anaerobic sludge extract, which allows us to obtain a C:N ratio equal to 80 and an iron concentration of 12 mg L^−1^, which, in contrast to the previous addition, allowed the stimulation of both biomass formation and lipid biosynthesis. Our results are consistent with those reported by a study that used heterotrophic nitrogen limitation strategies, where a C: N ratio higher than 12 promoted lipid biosynthesis in *Chlorella* sp. [35].

Generally, culturing strategies that promote both biomass and lipid production must improve the microalgae cultivation system efficiency. Anaerobic sludge extracts contain high concentrations of ammonium and phosphate [13]; thus, removing these nutrients using microalgae systems has been extensively studied. However, a study on the effect of iron supplementation during microalgae growth in anaerobic liquid digestate has shown that iron plays a significant role in the population dynamics of symbiotic life. It shows a positive effect on the synthesis of high-value products produced by microalgae, such as pigments, proteins, lipids, and carbohydrates [32]. 

Iron supplementation was shown to enable this by extending the exponential growth phase of microalgae cultures; simultaneously, increasing accumulated total lipids compared with non-supplemented cultures [36]. Thus, our study established a sludge extract bolus feeding strategy using respirometry to demonstrate the activation of oxygen-dependent metabolism after sludge addition. The nutritional contribution (2% *v v*^−1^) of the sludge extract simultaneously promoted biomass production and lipid biosynthesis compared to the previous addition of 0.5% *v v*^−1^ sludge, as seen in zone 4 (Figure 5).

## 4. Conclusions

Our study sheds light on the iron effect on the heterotrophic cultivation of *C. sorokiniana*, allowing us to obtain higher growth rates (0.17 h^−1^) during batch cultivation compared with those reported previously [4,5]. This finding was explained in terms of iron co-limitation by considering the kinetic effects of this phenomenon through mathematical modeling. Thus, the proposed model might provide a valuable tool for further research focused on the bioprocess design optimizing iron-based strategies to improve heterotrophic microalgae culturing performance.

Moreover, successful supplementation of anaerobic sludge extract to a nitrogen-deprived heterotrophic culture of *C. sorokiniana* was achieved. The addition of 2% *v v*^−1^ of sludge extract reactivated high oxygen-dependent cellular activity and promoted lipid biosynthesis, as it allowed the supplementation of 12 mg L^−1^ of iron and 50 mg TN L^−1^, alleviating nutritional deficiency. Therefore, iron-based supplementation strategies to produce lipids and other higher-value agal products might represent a sustainable alternative to manage industrial ADWS, and these require further developments at higher scales in order to assess the economic and environmental potential through techno-economic analysis (TEA) and life cycle analysis (LCA).

## Figures and Tables

**Table 1:** Experimental design for the quantification of respiratory activity on the heterotrophic growth of *C. sorokiniana*.

Treatment	Carbon Source (g L^−1^)	
	Acetate	Glucose
E1	0	5
E2	0	10
E3	5	5
E4	5	10

**Table 2:** Kinetic model parameters optimized from experimental data of iron supplementation effect during the late exponential phase of *C. sorokiniana* and iron-supplemented cultures of *Scenedesmus* sp. with reported data obtained from Ren et al. [22].

Parameter	Unit	*C. sorokiniana*	*Scenedesmus* sp.
*µ_Fe_*	h^−1^	0.58	0.105
*µ_max_*	h^−1^	0.256	0.085
*Y_x/s_*	g Biomass g Glucose^−1^	0.525	0.23
*Ks*	g Glucose L^−1^	5.33	12.56
*K_Fe_*	mg Fe L^−1^	12	0.0124
*Ki_Fe_*	mg Fe L^−1^	74	52
*n*		1	1.435

**Table 3:** The composition of anaerobically digested waste sludge supplied by Levapan S.A before and after pretreatment.

Elemental Analysis	Concentration (g L^−1^)	
	Anaerobic Waste Sludge	Anaerobic Sludge Extract
Total organic carbon (TOC.)	6.550	1.178
Total nitrogen (TN)	9.510	0.251
Iron (Fe)	11.670	0.060

## Data Availability

The data generated or analyzed during this study are included in this article, and the corresponding author could provide the primary data upon request.

## Supplementary Materials

The following are available online at https://www.mdpi.com/article/10.3390/bioengineering8060082/s1, Figure S1: Flowchart on the computational methodology for adequate use of iron co-limitation model. Figure S2: Specific growth rate increment calculated after iron addition (18–25 h) on the late exponential phase of the heterotrophic culture of C. sorokiniana. Table S1: mass balance equations for fed-batch simulations. The MATLAB code for ODE solution of the iron co-limitation model is available in https://github.com/ECharria/IronModelMicroalgae/tree/main (accessed on 23 May 2021).
