# Supplementary material for: Strategy for Managing Industrial Anaerobic Sludge through the Heterotrophic Cultivation of Chlorella sorokiniana: Effect of Iron Addition on Biomass and Lipid Production
Source: Bioengineering (Basel). 2021 Jun 10;8(6):82. doi: 10.3390/bioengineering8060082 (PMC8228024; doi:10.3390/bioengineering8060082)
Supplement: Supplementary file 1 [file bioengineering-08-00082-s001.zip › bioengineering-1216719-supplementary.pdf]

bioengineering

MDPI

Article

Strategy for Managing Industrial Anaerobic Sludge through the Heterotrophic Cultivation of *Chlorella sorokiniana*: Effect of Iron Addition on Biomass and Lipid Production

Esteban Charria-Girón [1], Vanessa Amazo [1], Daniela De Angulo [1], Eliana Hidalgo [1], María Francisca Villegas-Torres [2,3], Frank Baganz [4], and Nelson. H. Caicedo [1,3,*]

[1] Departamento de Ingeniería Bioquímica, Facultad de Ingeniería, Universidad Icesi, Calle 18 No. 122–135, 760031 Cali, Colombia; charria21@gmail.com (E.C.-G.); vaneaw_806@hotmail.com (V.A.); daniela.deangulo@gmail.com (D.D.A.); ely.hidalgo999@gmail.com (E.H.)
[2] Departamento de Ciencias Químicas, Facultad de Ciencias Naturales, Universidad Icesi, Calle 18 No. 122–135, 760031 Cali, Colombia; mfvillegas@icesi.edu.co
[3] Centro BioInc, Universidad Icesi, Calle 18 No. 122–135, 760031 Cali, Colombia
[4] Department of Biochemical Engineering, University College London, Gordon Street, London WC1H 0AH, UK; f.baganz@ucl.ac.uk
* Correspondence: nhcaicedo@icesi.edu.co; +57 318 754 8041

Supplementary Materials

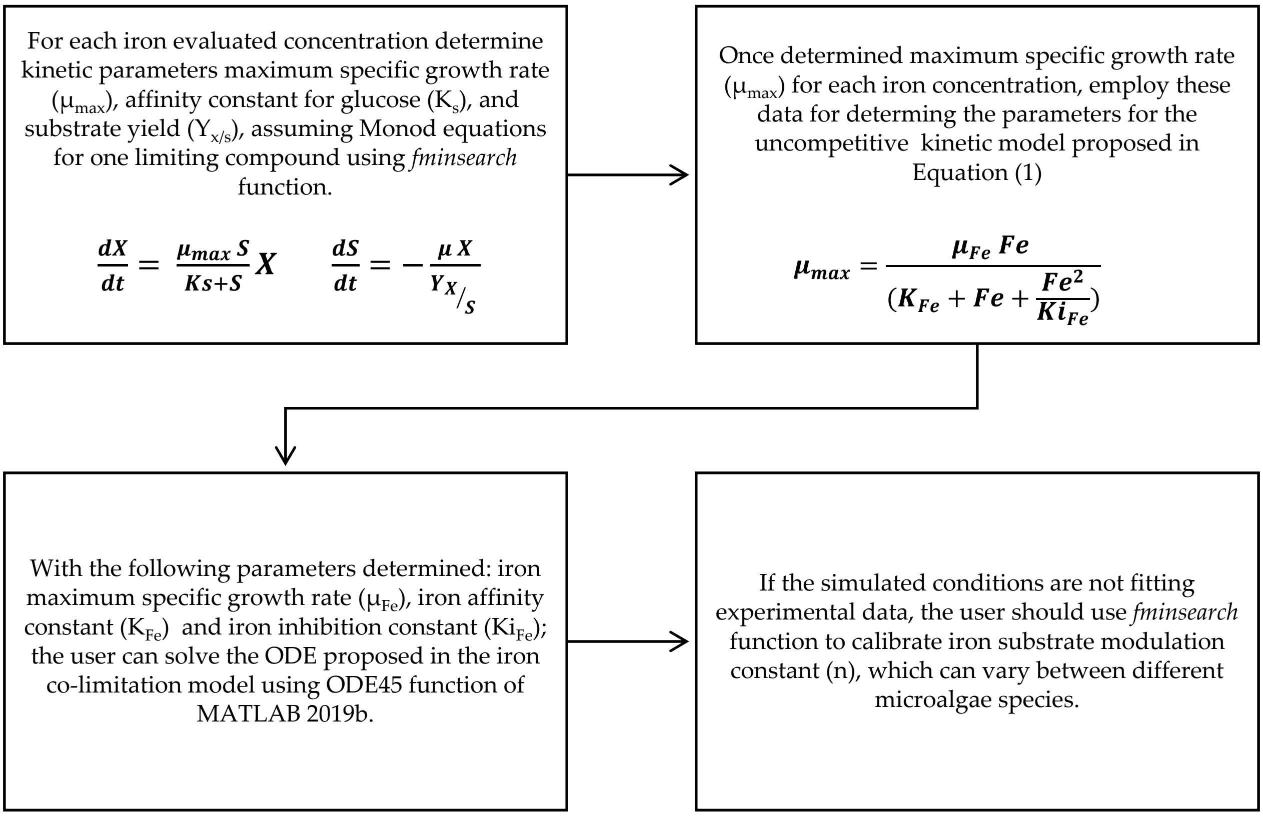

Figure S1. Flowchart on the computational methodology for adequate use of iron co-limitation model.

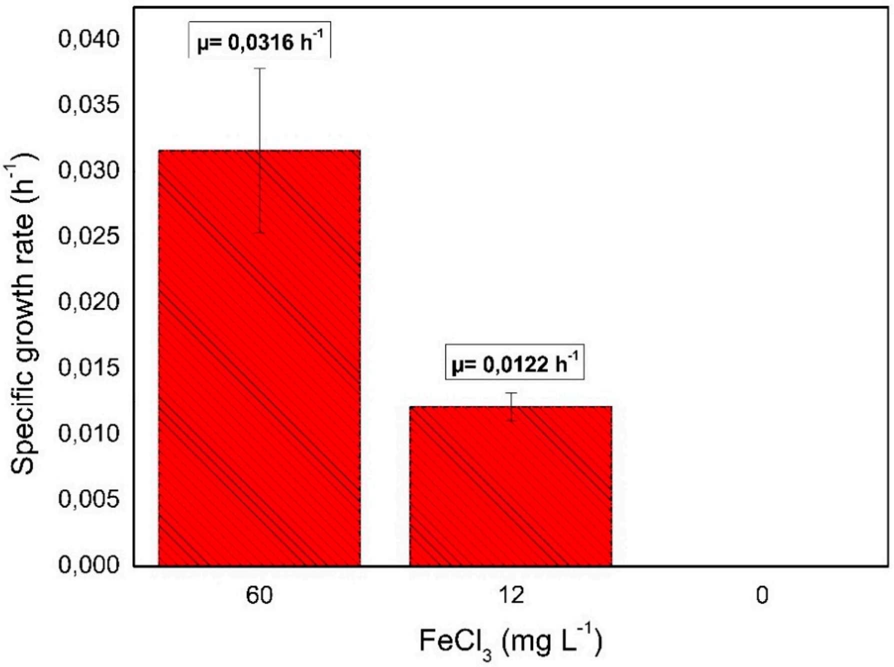

Figure S2. Specific growth rate increment calculated after iron addition (18–25 h) on the late exponential phase of the heterotrophic culture of *C.sorokiniana*.

Table S1. Mass balance equations for fed-batch bioaugmentation process; it is noting that substrate consumption for maintenance was assumed to be cero.

**Biomass growth mass balance**	$\frac{dX}{dt} = \left(\mu - \frac{Fin}{V}\right)X$
**Substrate consumption mass balance**	$\frac{dS}{dt} = \frac{-1}{Yxs}\mu X + (Sfeed * Fin - S)\frac{Fin}{V}$
**Volume balance**	$\frac{dV}{dt} = Fin = Fo\, e^{\mu_* t}$
**Initial flow rate**	$Fo = \frac{\frac{\mu_*}{Yxs}}{(Sfeed - S)} \quad \begin{cases} 6h \\ 0h \end{cases} ;\ \mu = 0.13\ h^{-1}$

Where X is the concentration of biomass (g L^{-1}); μ_* is the specific growth rate (h^{-1}) fixed during the fed-batch; Fin is the feeding rate (h^{-1}), for fed-batch cultivation; Fo is the initial feeding rate for exponential feeding profile (L min^{-1});V is the volume of the medium (L); Y_{XS} is the substrate yield coefficient (g g^{-1}); S is the glucose concentration (g L^{-1}) and Sin is the glucose concentration in the feed (g L^{-1}).

Supplementary Materials: Figure S1: Flowchart on the computational methodology for adequate use of iron co-limitation model. Figure S2: Specific growth rate increment calculated after iron addition (18–25 h) on the late exponential phase of the heterotrophic

culture of *C. sorokiniana*. Table S1: mass balance equations for fed-batch simulations. The MATLAB code for ODE solution of the iron co-limitation model is available in https://github.com/ECharria/IronModelMicroalgae/tree/main.

Author Contributions: Conceptualization, E. C.-G., E. H., M. F.V.-T., F. B., and N. H. C. O.; Formal analysis, E. C.-G., V. A., E. H., M. F. V.-T., and N. H. C. O.; Funding acquisition, N. H. C. O.; Investigation, E. C.-G., V. A., E. H., M. F. V.-T., and N. H. C. O.; Methodology, E. C.-G., V. A., E. H., M. F. V.-T., and N. H. C. O.; Project administration, N. H. C. O.; Resources, N. H. C. O.; Software, E. C.-G.; Supervision, M. F. V.-T., and N. H. C. O.; Visualization, E. C.-G., and D. D.; Writing—original draft, E. C.-G., V. A., and D. D.; Writing—review & editing, E. C.-G., D. D. and N. H. C. O. All authors have read and agreed to the published version of the manuscript.

Funding: COLCIENCIAS: grant number 275908943 funded this research.

Institutional Review Board Statement: Not applicable.

Informed Consent Statement: Not applicable.

Data Availability Statement: The data generated or analyzed during this study are included in this article, and the corresponding author could provide the primary data upon request.

Conflicts of Interest: The authors declare no conflict of interest. The funders had no role in the study's design; in the collection, analyses, or interpretation of data, in the writing of the manuscript, or in the decision to publish the results.
